# Polygenic risk scores for cardiovascular disease: clinical utility and limitations

**DOI:** 10.1186/s43044-026-00746-3

**Published:** 2026-05-18

**Authors:** Faith Adedayo Adejumo, Oluwayimika Oluwatobi Obielodan, Emmanuela Ojoagefu Egwu, Temitomi Jane Oyedele, Ebunoluwa Hannah Fasanye, Emmanuel Niyi-Oriolowo, Oluwadara Favour Odejayi, Olorundemilade Dunmade David, Temilade Adejumo, Fiyinfoluwa Adetoye, Nicholas Aderinto

**Affiliations:** 1 Bowen University, Iwo, Nigeria; 2 Ladoke Akintola University of Technology, Ogbomoso, Nigeria

**Keywords:** Cardiovascular diseases, Polygenic risk scores, Africa

## Abstract

**Background:**

Cardiovascular diseases (CVDs) are the leading cause of mortality worldwide, highlighting the need for improved risk prediction and prevention strategies. Polygenic Risk Scores (PRS), derived from genome-wide association studies (GWAS), offer a novel approach to estimating an individual’s genetic predisposition to conditions such as coronary artery disease (CAD), atrial fibrillation (AF), and heart failure (HF).

**Main text:**

PRS can improve early risk stratification, particularly among individuals with high genetic susceptibility who may not yet present traditional risk factors. Evidence suggests that PRS enhances risk prediction in younger populations and may inform targeted prevention efforts. However, several limitations constrain their clinical utility. These include limited predictive power relative to conventional risk models, a lack of diverse population representation in GWAS, and challenges in incorporating PRS into routine clinical workflows.

**Conclusions:**

While PRS holds promise for advancing personalized cardiovascular care, its clinical implementation requires overcoming key limitations. Increasing diversity in genetic research and integrating PRS with established clinical risk tools are critical steps toward realizing their full potential in CVD prevention and management.

## Introduction

Cardiovascular diseases (CVDs) have become a significant public health concern globally, with Africa facing a notable burden. Accounting for approximately 32% of all global deaths, CVDs represent the leading cause of mortality worldwide [1]. According to the World Health Organization, an estimated 17.7 million people succumbed to CVD in 2015, underlining the critical need for effective prevention and management strategies. CVDs primarily encompass coronary artery disease (CAD); cerebrovascular disease, including stroke and transient ischemic attack (TIA); peripheral artery disease (PAD), and aortic atherosclerosis [2].

Given the substantial heritability of CVD, advances in genetic research offer promising avenues for more precise risk prediction. Polygenic risk scores (PRS) leverage this genetic information to estimate an individual’s susceptibility to diseases like CVD by aggregating trait-associated alleles from genome-wide association studies (GWAS) [3, 4]. Developed initially in 2007, PRS for CVD has since evolved rapidly with the integration of low-cost genotyping, advanced imputation methods, and vast datasets representing diverse populations. Through GWAS, numerous genetic variants tied to key CVD risk factors such as smoking, renal dysfunction, elevated blood pressure, and abnormal lipid metabolism have been uncovered, offering deeper insights into individual disease susceptibility [4]. This review explores the clinical utility and limitations of PRS in CVD prediction and management, addressing current challenges and highlighting opportunities for broader implementation in clinical practice.

## Methodology

This narrative review synthesized evidence on the clinical utility, predictive performance, and limitations of polygenic risk scores (PRS) in cardiovascular disease (CVD). A comprehensive literature search was conducted in PubMed, Embase, Web of Science, Scopus, and Google Scholar for studies published up to July 2025. The search strategy included combinations of keywords and MeSH terms such as “polygenic risk score,” “cardiovascular disease,” “coronary artery disease,” “atrial fibrillation,” “heart failure,” “genome-wide association study,” “genetic risk prediction,” and “personalized medicine,” using Boolean operators to enhance sensitivity. Eligible studies included original research articles, cohort studies, case-control studies, randomized and post-hoc clinical analyses that evaluated PRS in cardiovascular disease risk prediction or clinical application. Studies focusing exclusively on monogenic cardiovascular disorders or that did not directly assess PRS utility were excluded. A total of 187 articles were identified, of which 43 studies met the inclusion criteria and were included in the final synthesis. A thematic synthesis approach was used to identify key domains, including predictive performance, clinical utility, implementation challenges, and population diversity.

## Development of polygenic risk scores

Polygenic Risk Scores (PRS) are quantitative tools that estimate an individual’s inherited susceptibility to disease by aggregating the effects of multiple genetic variants across the genome. These scores are typically calculated as the weighted sum of disease-associated alleles, with weights derived from genome-wide association studies (GWAS) [5]. Unlike rare monogenic mutations, which confer high risk but affect relatively few individuals, PRS capture the cumulative impact of hundreds to millions of common single-nucleotide polymorphisms (SNPs), each exerting a modest effect on disease risk [6].

The development of PRS began with simple additive models that summed risk alleles using GWAS-derived effect sizes. A pivotal discovery in this field was the identification of the *9p21* locus in 2007, which showed that each risk allele conferred a 25–30% increased risk of coronary artery disease (CAD) [7]. Since then, over 150 loci have been associated with CAD, implicating genetic pathways involved in lipid metabolism, vascular remodeling, and inflammation. Parallel discoveries have identified disease-associated loci for other cardiovascular conditions, including atrial fibrillation (AF), heart failure, and hypertension, enabling the construction of disease-specific PRS.

As methodological sophistication has increased, PRS construction has evolved from using only genome-wide significant SNPs to incorporating millions of variants through advanced algorithms. Modern approaches now employ linkage disequilibrium (LD)-aware methods such as LDpred and PRS-CS, along with Bayesian frameworks, to better estimate variant effect sizes and account for LD structure [8]. These genome-wide PRSs enable stratification of individuals along a continuum of genetic risk; however, the clinical interpretation of absolute risk thresholds remains uncertain and is not yet standardized for routine use.

The predictive performance of PRS is typically evaluated using metrics such as the area under the receiver operating characteristic curve (AUC), net reclassification improvement (NRI), and hazard ratios. Several studies have demonstrated that incorporating PRS into traditional clinical models significantly enhances risk stratification and prediction [9]. For instance, Khera et al. showed that PRS could identify 1.5% to 8% of the population at a threefold increased risk for diseases such as CAD, AF, type 2 diabetes, inflammatory bowel disease, and breast cancer [10].

**Fig. 1:**
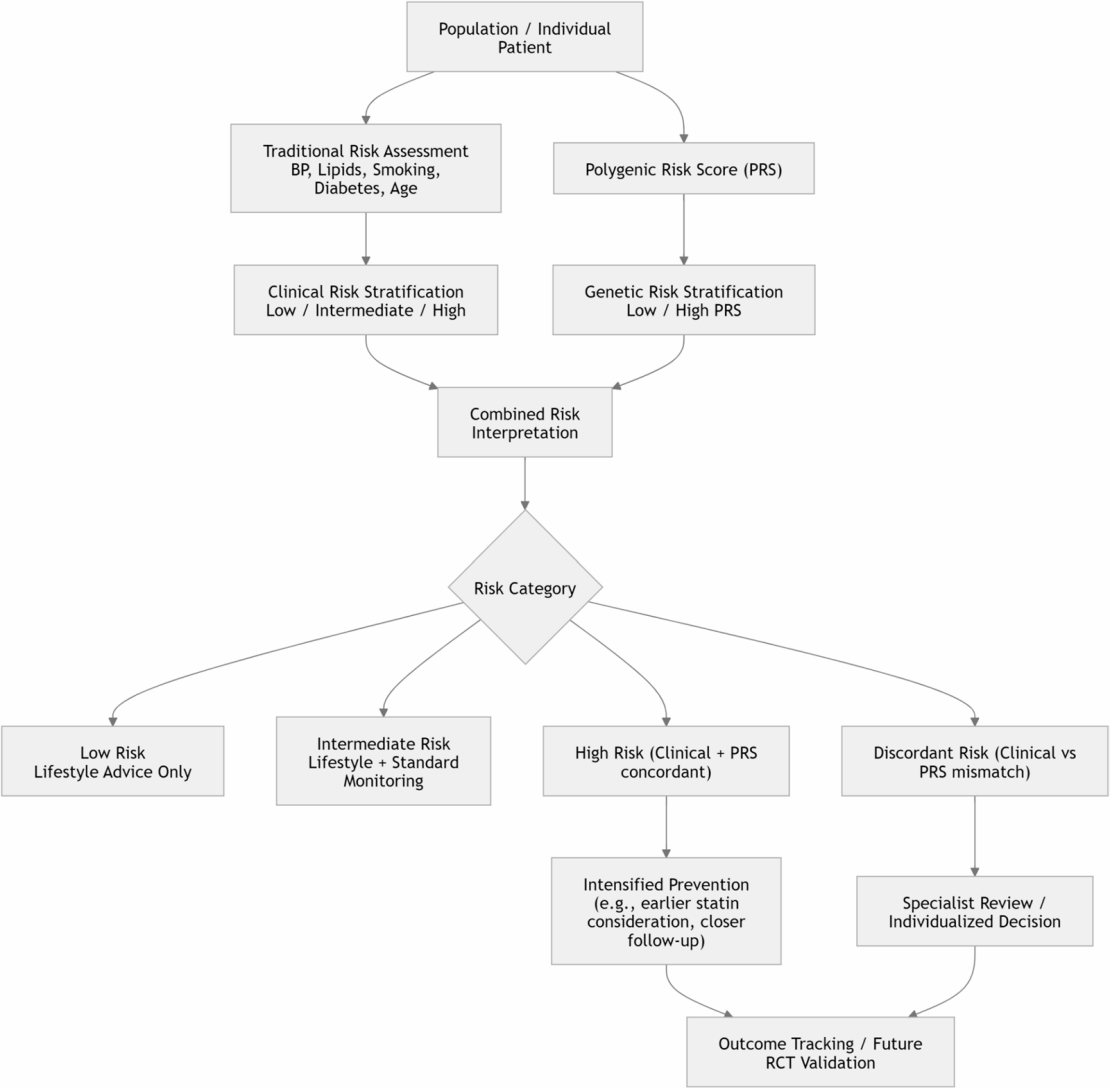
Conceptual framework for integration of polygenic risk scores with traditional clinical risk factors in cardiovascular disease risk stratification

Despite these advances, one of the most critical limitations of current PRS models is their limited applicability across diverse populations. Most GWAS and PRS development efforts have been conducted in individuals of European ancestry, which reduces predictive accuracy when applied to non-European populations due to differences in allele frequencies and LD structure [11, 12]. This disparity has prompted a growing push toward trans-ethnic PRS development and expanded representation of diverse ancestries in genetic studies to ensure equity and clinical utility across global populations (Fig. 1).

Furthermore, PRS accuracy may vary depending on disease stage and prior history. For example, Howe et al. found that PRS for CAD more effectively predicted incident events in individuals without prior disease, while discrimination was poorer in those with prevalent CAD. Similar patterns have been observed in atherosclerotic cardiovascular disease [13, 14].

As the field advances, PRSs are increasingly recognized as clinically actionable tools for personalizing prevention and treatment. Ongoing research is focused on optimizing their integration into routine care and ensuring equitable use across diverse patient populations.

### Interaction between polygenic and monogenic risk

An important and evolving area of cardiovascular genomics is the interaction between PRSs and rare monogenic variants, in which both forms of genetic variation jointly contribute to disease expression and clinical heterogeneity. Rather than operating in isolation, PRSs act as powerful phenotypic modifiers, altering penetrance, age of onset, and clinical severity in carriers of high-risk pathogenic variants. This interaction suggests a “two-hit” or additive model where the common genetic background (PRS) determines the clinical impact of a rare, high-effect mutation.

Evidence from atrial fibrillation (AF) indicates that rare variants and polygenic risk factors jointly determine disease susceptibility. In a large-scale study by Vad et al. utilizing exome sequencing data, rare predicted loss-of-function (pLOF) variants in multiple genes were associated with a significantly increased risk of incident AF [15]. Critically, the polygenic background modified the penetrance of these rare variants: carriers of pLOF variants with a high polygenic burden exhibited a substantially higher risk of AF and earlier disease onset than those with a low polygenic burden. Furthermore, this interaction extends to systemic outcomes; carriers of rare variants with a high PRS also demonstrate significantly elevated rates of cardiomyopathy and heart failure, indicating that the polygenic context shapes not only the susceptibility to AF but also the risk of its downstream complications. This highlights that monogenic testing alone is insufficient for precise risk stratification; the polygenic context is required to provide a complete prognostic profile.

A similar interaction has been described in hypertrophic cardiomyopathy (HCM), where the polygenic background modifies disease penetrance among individuals carrying pathogenic sarcomeric variants [16]. In this context, PRSs can stratify carriers into distinct risk trajectories: carriers with a high polygenic burden exhibit more severe phenotypic expression. In contrast, a lower polygenic burden may attenuate disease penetrance despite the presence of a pathogenic variant. These findings support a continuum model of genetic risk in which common and rare variants act additively to define an individual’s overall cardiovascular risk architecture.

Collectively, these insights suggest that PRSs should be viewed as complementary modifiers of monogenic risk rather than independent alternatives. Integrating both layers of genetic information may improve the identification of individuals most likely to develop disease or benefit from early screening. While these interactions are biologically robust, they require further validation in large, ancestrally diverse cohorts to establish standardized clinical thresholds for routine use.

### Critical appraisal of the evidence base for polygenic risk scores in cardiovascular disease

Across cardiovascular disease phenotypes, including coronary artery disease, atrial fibrillation, and heart failure, PRS demonstrate consistent and reproducible associations with incident outcomes and provide measurable stratification of inherited susceptibility. However, the current evidence base is best characterized by a strong statistical association rather than established clinical utility.

Most of the evidence arises from large observational cohort studies and population biobanks, particularly the UK Biobank and similar large-scale genomic resources, with relatively fewer case-control studies [11, 17]. Across study designs, PRS consistently identifies individuals at the extremes of genetic risk distribution who experience substantially elevated relative risk for cardiovascular outcomes such as myocardial infarction and atrial fibrillation [10, 18]. However, heterogeneity in study design, endpoint definitions, and population structure limits comparability across studies and complicates the synthesis of effect estimates.

In terms of predictive performance, PRS generally provides statistically significant improvement when added to conventional clinical risk models; however, the magnitude of improvement is modest. Most studies report incremental gains in discrimination metrics, such as AUC or C-index, of approximately 0.01–0.03, depending on baseline model performance and disease phenotype [5]. This pattern is consistent across cardiovascular conditions. In coronary artery disease, PRS identifies individuals at the extreme upper tail of genetic risk distribution with substantially increased relative risk but only modest improvement in overall risk discrimination at the population level [5, 10]. In atrial fibrillation, PRS improves stratification when integrated with clinical scores such as CHARGE-AF. Still, the added predictive value is largely driven by the separation of extreme risk groups rather than meaningful reclassification of intermediate-risk individuals [18–20]. Similarly, in heart failure, polygenic risk scores are associated with incident disease and hospitalization outcomes across multiple cohorts; however, their incremental improvement beyond established clinical predictors appears modest and varies across study designs [17, 21, 22].

There is also substantial heterogeneity across studies in both methodology and population characteristics. Most PRS models are derived from individuals of European ancestry, limiting external validity in non-European populations due to differences in allele frequency, linkage disequilibrium structure, and genetic architecture [9, 12, 23, 24]. This ancestry-related performance gap reduces predictive accuracy in underrepresented populations and raises important concerns regarding the accuracy of risk stratification.

PRS represents robust and biologically informative markers of inherited cardiovascular susceptibility. However, their clinical translation is currently constrained by modest incremental gains in predictive performance, heterogeneity in study populations, and limited generalizability beyond European-ancestry cohorts. While PRS may enhance risk stratification at the extremes of the genetic risk distribution, its role in routine cardiovascular practice remains investigational. It should be considered an adjunct to established clinical risk assessment tools.

A critical appraisal of key studies evaluating PRS in cardiovascular disease is provided in Table 1, focusing on study design, effect sizes, and clinical relevance.

**Table 1:** Critical appraisal of representative PRS studies: strength of evidence and clinical relevance

S/*N*	Author (Year)	Study design	Key metric reported	Magnitude of improvement	Nature of evidence	Key limitation	Clinical interpretation
1	Khera et al. (2022)	Prospective cohort	HR (~3-fold risk)	Small ΔAUC	Observational	European ancestry bias	Strong association but limited clinical reclassification impact
2	Khera et al. (2019)	Case-control	OR 3.7	No discrimination metric	Observational	Selected early-onset cohort	High relative risk; unclear population-level utility
3	Al Rifai et al. (2022)	Prospective cohort	Event prediction	Modest improvement	Observational	Subgroup-specific (CAC = 0)	May refine risk in niche populations
4	Hagström et al. (2022)	Post-hoc RCT	Relative risk reduction	Not designed for prediction	Post-hoc trial analysis	Secondary analysis	Suggests differential treatment benefit; not causal evidence
5	Viigimaa et al. (2022)	RCT	Behavioral outcomes	Not applicable	Interventional	Small sample	Affects behavior, not hard outcomes
6	Oni-Orisan et al. (2022)	Cohort	HR (0.41 statin benefit)	Not reported	Observational	No hard endpoints	Suggests pharmacogenomic potential
7	Landen et al. (2025)	Prospective cohort	AUC, NRI	ΔAUC ~0.01–0.03; NRI 7–14%	Observational	European ancestry	Statistically significant but modest clinical gain
8	Hart et al. (2022)	Cohort	C-index	Moderate increase	Observational	Outcome definition limitations	Improved prediction; uncertain impact on decisions
9	Paranjpe et al. (2021)	Prospective cohort	OR ~2	Modest improvement	Observational	CAD-only population	Predictive but not actionable
10	Han et al. (2023)	Cohort	AUC	Large improvement	Observational	Single-center	Likely overestimation; limited generalizability
11	Ahn HJ et al. (2025)	Prospective cohort	NRI	~13% improvement	Observational	European-only	Incremental improvement; modest
12	Lanfear et al. (2020)	Trial-derived cohort	HR 1.17	Not reported	Observational	Not primary endpoint	Early pharmacogenomic signa

## Clinical utility of polygenic risk scores in cardiovascular diseases

Polygenic risk scores (PRS) are an emerging research tool for cardiovascular risk stratification. While they provide additional information on inherited susceptibility to cardiovascular disease, their clinical utility remains under active evaluation and has not yet been established in routine practice. By analyzing genetic data, PRS can provide insights into an individual’s predisposition to cardiovascular conditions such as coronary artery disease (CAD), atrial fibrillation (AF), heart failure, stroke, and others.

Although there are currently no official guidelines for the use of polygenic risk scores (PRS) in clinical practice, recent studies have demonstrated promising potential for their use in the management, especially the prevention, of various cardiovascular diseases.

### Coronary artery disease

Polygenic Risk Scores (PRS) have shown potential utility in risk prediction and stratification for coronary artery disease (CAD), although their clinical impact remains modest and largely observational. Marston et al., in a large UK Biobank cohort involving 330,201 participants, demonstrated that a CAD polygenic risk score was associated with increased myocardial infarction (MI) risk, particularly in adults under 50 years of age, and enabled risk reclassification into higher genetic risk categories. However, the clinical implications of such reclassification, including earlier statin initiation, remain observational and are not supported by guidelines [18].

Similarly, a genetic case-control study by Khera et al. involving 2,081 early-onset MI patients found that a high PRS conferred a 3.7-fold increased risk of MI, surpassing the predictive power of familial hypercholesterolemia [25].

The utility of PRS across diverse populations was further supported by Al Rifai et al. in the MESA study, which included 3,132 subjects, where PRS predicted ASCVD events among women and non-White participants with zero coronary artery calcium. In a secondary prevention setting, Hagström et al. found that high-PRS patients had higher absolute MACE rates and derived greater benefit from alirocumab therapy than placebo [26, 27].

Intervention-focused studies have also shown associations between PRS and treatment response. In a randomized trial by Viigimaa et al., disclosure of high PRS alongside genetic counseling was associated with increased statin uptake and improved lipid profiles. Oni-Orisan et al. demonstrated that individuals with high PRS experienced greater relative benefit from statin therapy in a pharmacogenomic cohort, although these findings remain exploratory [28, 29].

Overall, current evidence suggests that PRS adds a meaningful layer of insight into inherited susceptibility to coronary artery disease, particularly in identifying individuals at higher lifetime risk whom traditional short-term risk models may not capture. This may be especially relevant in younger populations, where early preventive strategies could have the greatest long-term impact. However, translating this additional risk information into specific clinical actions remains uncertain, and PRS is not currently incorporated into routine decision-making or guideline-directed care. At present, its role is best considered as complementary to established risk assessment approaches, with clearer clinical applications likely to emerge as prospective evidence evolves.

### Atrial fibrillation (AF)

Polygenic Risk Scores (PRS) have demonstrated moderate predictive ability in identifying individuals at increased risk of atrial fibrillation (AF), both independently and when integrated with established clinical models. In a large prospective cohort by Marston et al., involving over 36,000 participants, PRS was associated with increased AF risk per 1-SD increase. Those in the highest PRS quintile had more than twice the risk of AF compared with those in the lowest quintile, with improved predictive performance when combined with CHARGE-AF and NT-proBNP [18].

Segan et al. evaluated over 285,000 individuals and found that PRS modestly improved risk discrimination when incorporated into CHA₂DS₂-VASc and HARMS₂-AF models, yielding small improvements in AUC and reclassification indices. Similarly, Noor et al. showed that PRS stratification in low clinical risk AF patients (CHA₂DS₂-VA ≤ 1) was associated with cardiovascular outcomes, suggesting potential stratification value in selected subgroups [19, 20].

Hart et al. focused on stroke and systemic embolism (SSE) risk and showed that PRS improved predictive accuracy (C-index increased from 0.66 to 0.72 for SSE), particularly in older adults [20]. However, AF diagnosis in this study was inferred from stroke events, which may limit specificity.

Vogel et al. conducted a cohort study showing that higher PRS was associated with increased AF recurrence after cardioversion. Despite the small sample size, these findings are hypothesis-generating and require further validation in larger trials [30].

These findings indicate that PRS can modestly refine risk estimation in atrial fibrillation, particularly when integrated with existing clinical prediction tools. Its potential value may lie in identifying individuals at the margins of conventional risk categories, where decisions around monitoring or screening are less clear-cut. However, the overall magnitude of improvement remains limited, and it is not yet evident that PRS-informed risk stratification leads to different clinical decisions or improved outcomes. As such, PRS currently plays a supportive role in risk assessment, with its clinical relevance pending further validation in prospective and interventional settings.

### Heart failure

Recent studies suggest that polygenic risk scores (PRS) may modestly improve risk prediction for heart failure (HF), although findings are heterogeneous and largely observational.

In a large prospective cohort, Paranjpe et al. developed and externally validated an ischemic HF-PRS in patients with coronary artery disease (CAD). Individuals in the top PRS decile had approximately a twofold increased risk of developing ischemic HF, with consistent results across biobanks, although applicability was limited to European populations [17].

Similarly, Han et al. applied a 69-SNP PRS in a cohort of HF with preserved ejection fraction (HFpEF) patients, showing that individuals with high PRS had increased one-year mortality risk and improved model performance compared with clinical predictors (AUC 0.852 vs. 0.696) [21]. However, this was derived from a single-center Han Chinese population, limiting generalizability.

In broader population-level studies, Ahn HJ et al. used LDpred2-based PRS in approximately 500,000 individuals. They found an increased risk of incident HF among individuals with high PRS, with modest net reclassification improvement when added to clinical models [22]. Lanfear et al. developed and externally validated a polygenic score for β-blocker response in heart failure using large clinical cohorts, including data from the UK Biobank (approximately 59,000 participants) and independent heart failure cohorts. The study demonstrated that genetic stratification could identify patients who derive differential survival benefit from β-blocker therapy, with higher polygenic risk scores being associated with improved treatment response and survival outcomes in selected subgroups [31]. These findings highlight the potential for PRS to move beyond risk prediction toward pharmacogenomic stratification, although clinical implementation remains exploratory and not yet guideline-directed.

### Emerging evidence of prs in other cardiovascular diseases

#### Venous thromboembolism (VTE)

Recent large-scale genome-wide association analyses have substantially advanced understanding of the genetic architecture of venous thromboembolism (VTE). In a multi-cohort study including over 81,000 cases and more than 1.4 million controls, 93 genetic risk loci were identified, of which the majority were novel and enriched in biological pathways related to coagulation and platelet function. These findings reinforce the central role of hemostatic regulation in VTE susceptibility [32].

A genome-wide polygenic risk score derived from these loci demonstrated strong stratification of VTE risk across the population distribution. Individuals in the top 0.1% of the PRS distribution exhibited VTE risk comparable to carriers of high-impact monogenic thrombophilic variants such as F2 G20210A and F5 p.R534Q, highlighting that extreme polygenic burden may approximate monogenic-level risk. Conversely, carriers of these monogenic mutations within the lowest 10% of the PRS distribution exhibited risk levels similar to those of the general population, demonstrating meaningful modulation of monogenic risk by the polygenic background [32].

Importantly, the VTE PRS improved risk prediction beyond both established genetic variants and conventional clinical risk factors, supporting its additive predictive value. Mendelian randomization analyses further demonstrated partial overlap between the architectures of arterial and venous thrombosis, with body mass index and smoking showing concordant directional effects on both conditions. In contrast, systolic blood pressure and triglyceride levels showed discordant associations. These findings suggest that VTE shares both overlapping and distinct biological determinants compared with atherosclerotic cardiovascular disease, reinforcing the need for phenotype-specific risk prediction models.

#### Stroke

Cross-ancestry GWAS meta-analyses of over 110,000 stroke cases have identified 89 independent loci, substantially expanding known genetic architecture. Integrative polygenic risk scores incorporating multi-ancestry and vascular trait data improve prediction of ischemic stroke across European, African, and East Asian populations. Importantly, these PRS models remain independently predictive even after adjustment for clinical risk factors in large cardiometabolic cohorts, supporting their role as complementary tools rather than replacements for established risk models [33].

#### Aortic stenosis

Large-scale analyses from population cohorts and clinical trial datasets have enabled the development of a genome-wide polygenic risk score comprising over five million variants. This score is significantly associated with incident aortic stenosis across independent cohorts, with effect sizes comparable to several established clinical risk factors [34].

However, adding the PRS to clinical models results in only modest improvement in discrimination (C-index increase of ~ 0.01–0.02), indicating limited incremental predictive performance beyond age and traditional cardiovascular risk factors. Despite these modest improvements in discrimination, the aortic stenosis PRS still provides incremental value as a risk stratification tool, particularly in supporting earlier identification of individuals at higher genetic susceptibility when combined with clinical risk factors [34].

#### Aortic aneurysm

Abdominal aortic aneurysm (AAA) has a substantial heritable component and shares partial genetic architecture with other cardiovascular diseases, suggesting a potential role for polygenic risk stratification in screening strategies. A polygenic risk score (PRS) developed using pleiotropy-informed meta-analyses of large-scale genetic datasets demonstrated improved predictive performance compared with AAA-specific models alone, with a 22.7% increase in explained variance [35].

Individuals in the intermediate and high PRS tertiles showed significantly increased risk of AAA compared with the low-risk group, with hazard ratios of 2.13 (95% CI 1.61–2.82) and 3.70 (95% CI 2.86–4.80), respectively, after adjustment for clinical risk factors. Simulation-based analyses further suggest that integrating genetic risk with smoking status could improve the efficiency of screening strategies, potentially enabling earlier identification of high-risk individuals and more targeted surveillance approaches [35].

Despite these promising findings, current evidence remains largely model-based, and the lack of prospective validation and integration into established screening programs limits clinical implementation. Nevertheless, these results highlight the potential for PRS to refine AAA screening strategies when combined with conventional risk factors such as smoking history.

A comparative summary of the predictive performance, study design, and clinical interpretation of PRS across major cardiovascular diseases is presented in Table 2.

**Table 2:** Comparative summary of study design, predictive performance, and clinical interpretation of polygenic risk scores across cardiovascular diseases

S/*N*	Cardiovascular disease	Study design type	Population source	Predictive performance	Effect size	Clinical interpretation
1	Coronary artery disease (CAD)	Large cohort studies, biobank analyses	Predominatly European ancestry	Modest improvement in discrimination when added to clinical models	Higher risk in top PRS percentiles (≈2–3-fold)	Improves risk stratification, especially in younger individuals; limited impact on clinical decision-making
2	Atrial fibrillation (AF)	Cohort studies + post-hoc RCT analyses (e.g., LOOP)	Mixed ancestry (mostly European)	Modest incremental predictive value	Increased risk per SD increase in PRS	May identify high-risk individuals; no established role in guiding therapy
3	Heart failure (HF)	Cohort and GWAS-based studies	European, Asians	Modest improvement in prediction	Increased risk across PRS gradients	Predictive association present; clinical thresholds not defined
4	Stroke	Multi-ancestry GWAS and cohort data	Multi-ancestry	Modest improvement when combined with clinical scores	Moderate risk increase	Adds value when integrated with traditional risk factors; not a standalone tool
5	Venous thromboembolism (VTE)	GWAS and polygenic modelling studies	European, African, East Asians	Stronger stratification at extremes	Higher relative risk at high PRS	Potential for risk stratification; not clinically implemented
6	Aortic stenosis	Population cohort studies	European ancestry dominant	Modest improvement in prediction	Moderate risk increase	Early identification possible; limited impact on management
7	Abdominal aortic aneurysm (AAA)	GWAS and modeling studies	Euopean ancestry	Improvement in model performance	Risk varies across PRS categories	Potential role in screening refinement; not clinically validated

### Comparison with traditional risk factors

Polygenic Risk Scores (PRS) and traditional risk factors, such as the Framingham Risk Score (FRS), are both used to assess cardiovascular disease (CVD) risk but through different mechanisms. FRS estimates the 10-year risk based on age, sex, cholesterol levels, blood pressure, diabetes, and smoking status. In contrast, PRS calculates genetic predisposition by aggregating the effects of multiple genetic variants identified through genome-wide association studies (GWAS) [11]. PRS provides additional risk information beyond what is captured by clinical models like FRS. For example, PRS can identify individuals at high genetic risk for coronary artery disease (CAD) who are classified as low or intermediate risk according to FRS, particularly in younger individuals. PRS offers a lifetime risk perspective, as genetic predisposition remains constant, unlike FRS, which focuses on short-term risk and may not predict long-term outcomes effectively [5].

Integrating PRS with traditional risk factors has been shown to improve overall risk prediction accuracy, especially in intermediate-risk individuals, according to FRS. While PRS adds valuable insights, its predictive value is modest compared to that of traditional risk factors when used alone. The incremental benefit of PRS is notable when combined with traditional risk scores, enhancing stratification and informing interventions such as lifestyle changes or statin therapy [5].

### Risk stratification and role in personalized medicine

Polygenic Risk Scores (PRS) enhance cardiovascular disease (CVD) risk stratification by categorizing individuals into low-, intermediate-, and high-genetic risk groups. While PRS improves risk prediction beyond traditional clinical factors, the magnitude of improvement is generally modest, and no validated thresholds for clinical implementation are currently available.

For example, a high CAD PRS has been associated with a two- to threefold increased risk of coronary artery disease, supporting its role in identifying individuals with elevated inherited susceptibility [10]. However, current evidence does not support changes in clinical management such as earlier statin initiation, increased screening frequency, or treatment escalation based solely on PRS categories. In particular, intermediate PRS has no established clinical implications.

Although PRS-based stratification may in the future support earlier preventive strategies in high-risk individuals, such applications remain hypothetical and have not been validated in prospective interventional studies. At present, most evidence is derived from observational cohorts, limiting translation into routine clinical decision-making.

PRS may also contribute to personalized pharmacotherapy strategies. High genetic risk has been associated with differential benefit from statin therapy, with greater absolute treatment effects observed in higher-risk groups in exploratory analyses [10]. However, these findings remain hypothesis-generating and are not supported by randomized clinical trial evidence or incorporated into current guideline recommendations.

Overall, PRS improves cardiovascular risk stratification at a population level, but its clinical utility remains limited. It should currently be viewed as a research tool for risk enrichment rather than a basis for routine clinical decision-making.

### Evidence from clinical and implementation trials of polygenic risk scores

A key limitation to the clinical implementation of polygenic risk scores (PRS) is the lack of robust randomized clinical trial evidence demonstrating improved cardiovascular outcomes. Most available data come from observational studies or post hoc analyses of completed trials, with relatively few prospective implementation studies.

Evidence from post-hoc analyses of randomized trials suggests that PRS may help refine risk stratification and identify subgroups with differential benefit from preventive interventions. In a prespecified post hoc analysis of the LOOP randomized trial involving 5,656 AF-naive individuals aged ≥ 70 years, AF polygenic risk scores (PRSAF) were evaluated in relation to outcomes following implantable loop recorder (ILR) screening versus usual care. The study demonstrated that ILR screening reduced stroke or systemic embolism in individuals with higher PRS (HR 0.65; 95% CI 0.43–0.97), but not in those with lower PRS (HR 1.06; 95% CI 0.72–1.57), with a significant interaction between screening strategy and genetic risk (Pinteraction = 0.006). However, these findings remain hypothesis-generating, are limited by post-randomization subgroup analyses and the lack of pre-specified clinical decision thresholds, and do not establish the clinical benefit of PRS-guided screening [36].

In contrast to post-hoc evidence, prospective implementation trials are beginning to evaluate the real-world integration of PRS into clinical care pathways. The GenoVA study is a pragmatic randomized controlled trial designed to assess the implementation of PRS in primary care settings. This study evaluates the processes of genetic testing, reporting of PRS results to patients and primary care providers, and incorporation of genetic risk information into clinical decision-making. The trial focuses on several common cardiovascular and metabolic conditions, including coronary artery disease, atrial fibrillation, type 2 diabetes, and selected cancers. Key endpoints include time to new diagnosis, uptake of preventive therapies, and clinician engagement with genetic risk information. Importantly, the GenoVA study also incorporates strategies to address equity in PRS implementation by improving recruitment of underrepresented populations and evaluating ancestry-informed risk interpretation [37]. However, this study primarily evaluates feasibility and implementation outcomes and does not assess hard cardiovascular endpoints such as myocardial infarction, stroke, or cardiovascular mortality.

Despite these advances, no large-scale randomized clinical trial has yet demonstrated that PRS-guided care improves hard cardiovascular outcomes compared with standard risk assessment. Current studies also vary widely in endpoints, ranging from changes in risk factor management to disease incidence, limiting comparability across studies.

Future trials should therefore adopt pragmatic, health-system–embedded randomized designs with clearly defined PRS risk thresholds, standardized clinical action pathways, and hard cardiovascular endpoints such as myocardial infarction, stroke, and cardiovascular mortality. In addition, integration with electronic health record systems, cost-effectiveness evaluation, and stratified analyses across ancestry groups will be essential to determine scalability and equitable clinical implementation.

Overall, while early post-hoc and implementation studies suggest potential clinical relevance, robust randomized evidence demonstrating outcome benefit remains the critical missing step before routine clinical adoption of PRS.

### Current guideline position on polygenic risk scores in cardiovascular disease

Despite growing evidence linking PRS with CVD outcomes, PRS is not currently incorporated into major clinical practice guidelines for cardiovascular risk assessment or prevention. Contemporary recommendations from major cardiovascular societies, including the European Society of Cardiology (ESC), the American Heart Association/American College of Cardiology (AHA/ACC), and the National Institute for Health and Care Excellence (NICE), continue to endorse risk stratification based on established clinical factors such as age, sex, blood pressure, lipid profile, smoking status, and diabetes mellitus. None of these guideline frameworks currently recommend the use of PRS for routine risk prediction or for guiding pharmacological interventions in primary or secondary prevention settings [38–40].

This conservative position is driven by the fact that statistically significant associations have not yet translated into validated clinical decision thresholds that would support changes in management strategies, such as the initiation of statin therapy or anticoagulation based solely on genetic risk stratification. While PRS improves discrimination metrics such as C-index and AUC, the magnitude of improvement remains generally modest [10, 11].

Furthermore, major societies emphasize that the current evidence base lacks the interventional validation required for clinical adoption. As detailed earlier, there is a notable absence of large-scale randomized controlled trials demonstrating that PRS-guided decision-making improves hard cardiovascular outcomes, such as myocardial infarction or mortality [23, 41]. Additionally, the lack of ancestral diversity in model development remains a significant barrier to guideline endorsement due to concerns regarding misclassification and inequitable application across diverse populations [9, 12] (Table 3).

**Table 3:** Limitations and challenges of PRS

Limitation	Description	Clinical implication
Lack of Ancestral Diversity	~83.8% of GWAS participants are of European ancestry, reducing PRS accuracy in non-European populations due to differences in allele frequencies and LD patterns.	May exacerbate health disparities; limited applicability in diverse populations.
Modest Predictive Power	Traditional risk models (AUC 80–85%) outperform PRS alone; PRS adds ~11.7% improvement to models like QRISK2.	Limited standalone utility; best used as a complementary tool.
Gene-Environment Interactions	PRS does not account for dynamic lifestyle, dietary, or socioeconomic factors.	May miss environmental contributions to CVD risk.
Ethical and Psychosocial Concerns	Risk of genetic determinism, misinterpretation, or discriminatory use; DTC testing lacks counseling.	Requires genetic counseling and clinician education to ensure responsible use.
Infrastructure Gaps	Limited genomic research capacity in low- and middle-income countries; PRS primarily accessible via DTC or private hospitals.	Restricts equitable access, particularly in resource-limited settings.

Current guideline positions reflect the translational gap between statistical risk prediction and clinically actionable decision-making. At present, PRS is regarded by these bodies as an emerging research tool rather than a standalone clinical instrument for cardiovascular prevention strategies. Future incorporation into formal recommendations will depend on evidence of cost-effectiveness, standardized risk thresholds, and improved patient outcomes demonstrated through robust clinical trials.

## Barriers to clinical implementation and translational positioning of polygenic risk scores in cardiovascular disease

PRS have demonstrated consistent associations with CVD outcomes across multiple cohorts; however, their translation into routine clinical practice remains limited by a combination of operational, economic, and health system–level barriers. These challenges extend beyond predictive performance and reflect a broader gap between statistical risk estimation and actionable clinical utility.

A primary hurdle is the lack of standardized clinical workflows for integrating PRS into care pathways. Unlike traditional risk scores embedded within established tools, PRS is not routinely incorporated into electronic health records or clinical decision-support systems, limiting its accessibility and scalability in real-world settings [20]. The absence of validated clinical decision thresholds compounds this. At the same time, PRS provides a continuous spectrum of risk; there are no universally accepted cut-offs linked to specific interventions, such as statin initiation or anticoagulation [42].

Uncertainty also persists regarding clinical responsibility for interpretation and follow-up. It remains unclear who should manage PRS results: cardiologists, primary care physicians, or clinical geneticists. This ambiguity, combined with limited genomic literacy among frontline clinicians, represents a significant practical barrier to widespread implementation. Furthermore, economic considerations constrain adoption; while genotyping costs have decreased, the cost-effectiveness of PRS relative to existing low-cost clinical risk models remains unproven [12]. Additional resource requirements for bioinformatics infrastructure and genetic counseling may further limit scalability, particularly in resource-constrained settings, potentially widening global health inequities [12, 43].

Psychosocial and ethical challenges also persist, as PRS results may be misinterpreted as deterministic risk estimates, potentially leading to patient anxiety or false reassurance. The absence of standardized counseling frameworks further complicates safe implementation, particularly in direct-to-consumer contexts.

These operational barriers are further reinforced by the current lack of ancestral diversity in genetic models, the absence of randomized controlled trial evidence demonstrating outcome benefit, and the resulting lack of formal guideline endorsement [9, 11, 12, 38–40]. Addressing these systemic issues will require coordinated efforts to develop clear clinical pathways and integrate PRS into existing healthcare infrastructures.

## Future directions and research gaps

PRS are increasingly recognized as promising tools in predictive medicine, with expanding applications in cardiovascular risk stratification and preventive genomics. While their potential to refine risk prediction is widely acknowledged, their clinical translation remains constrained by limitations in predictive performance, population generalizability, and real-world implementation pathways. Moving forward, progress will depend on methodological refinement and the development of clear frameworks for clinical integration.

A major and persistent priority is resolving the reduced transferability of PRS across ancestries. Because most participants in genome-wide association studies (GWAS) are of European ancestry, predictive performance is frequently attenuated in underrepresented populations [2, 8]. This gap not only limits generalizability but may also exacerbate existing healthcare disparities. Large-scale initiatives such as the All of Us Research Program and Biobank Japan are currently addressing this by expanding genomic diversity and improving the representativeness of reference datasets [44, 45]. The continued expansion of multi-ancestry GWAS datasets is essential for developing equitable and globally applicable PRS models.

Another vital area for development is the evolution of PRS from isolated genetic markers into broader multi-modal risk frameworks. Integrating genetic data with proteomic, metabolomic, and epigenetic markers (such as methylation-based scores) has already shown promise in enhancing predictive accuracy beyond conventional clinical models [4]. Such systems-level approaches may enable earlier identification of high-risk individuals and support personalized preventive strategies that account for both inherited susceptibility and environmental exposures [4].

Future progress must also focus on the evidence required to justify clinical uptake. While PRS improves statistical risk prediction, there is limited data demonstrating cost-effectiveness relative to existing low-cost clinical tools. As discussed in Sect.  4.7, outcome-driven studies and randomized trials are needed to establish clinically meaningful risk thresholds defined cut-offs that can directly inform interventions such as statin initiation or anticoagulation.

The long-term success of PRS will depend on system-level foundations, including robust health policy, clinician education in genomic literacy, and the integration of genomic data into automated electronic health record workflows. By coordinating advances in genomics with health economics and clinical informatics, PRS can transition from an investigational research tool into a responsible and effective instrument for cardiovascular prevention.

## Conclusion

Polygenic risk scores are a biologically informative and statistically robust approach to quantifying inherited susceptibility to cardiovascular disease. Across multiple cardiovascular phenotypes, PRS improves risk stratification when added to clinical models; however, the magnitude of improvement is generally modest, and these statistically significant associations have not consistently translated into clinically meaningful changes in decision-making.

Current evidence is derived predominantly from observational cohort studies, with limited randomized trial data demonstrating clinical benefits. In addition, the absence of standardized risk thresholds, limited ancestral diversity, and lack of guideline endorsement currently restrict clinical implementation.

Overall, PRS should be viewed as an adjunctive tool for risk stratification and population enrichment rather than a standalone strategy for routine cardiovascular risk assessment. At present, polygenic risk scores are not ready for routine clinical deployment in cardiovascular medicine. Future clinical adoption will depend on robust interventional evidence, standardized implementation frameworks, improved population diversity, and demonstrated cost-effectiveness in real-world healthcare systems.

## Data Availability

No datasets were generated or analysed during the current study.

## Abbreviations

CVD: Cardiovascular sisease
PRS: Polygenic risk score
GWAS: Genome-Wide Association Study
SNP: Single-nucleotide polymorphism
LD: Linkage disequilibrium
CAD: Coronary artery disease
AF: Atrial fibrillation
HF: Heart failure
HFpEF: Heart failure with preserved ejection fraction
VTE: Venous thromboembolism
AAA: Abdominal aortic aneurysm
AS: Aortic stenosis
MI: Myocardial infarction
SSE: Stroke and systemic embolism
ASCVD: Atherosclerotic cardiovascular disease
PAD: Peripheral artery disease
TIA: Transient ischemic attack
FRS: Framingham risk score
CHARGE-AF: Cohorts for heart and aging research in genomic epidemiology–atrial fibrillation
CHA₂DS₂-VASc: Congestive heart failure, hypertension, age ≥ 75, diabetes, stroke/transient ischemic attack, vascular disease, age 65–74, sex category
HARMS₂-AF: Hypertension, age, race, male sex, smoking, alcohol, family history–atrial fibrillation
NT-proBNP: N-terminal pro-B-type natriuretic peptide
AUC: Area under the receiver operating characteristic curve
NRI: Net reclassification improvement
HR: Hazard ratio
CI: Confidence interval
SD: Standard deviation
C-index: Concordance index
EHR: Electronic health record
RCT: Randomized controlled trial
ILR: Implantable loop recorder
BMI: Body mass index
DTC: Direct-to-consumer
HCM: Hypertrophic cardiomyopathy
PRS-AF: Atrial fibrillation polygenic risk score
